# Mechanical Valve Dysfunction in Yemen

**DOI:** 10.4103/1995-705X.73207

**Published:** 2010

**Authors:** A. Raboi, A. Al-Motarreb, A. Al-Kanadi, A.A. Abdulmughni, A. Kadi

**Affiliations:** Cardiac Centre, Al-Thawrah Hospital, Faculty of Medicine, Sana’a University, Yemen

**Keywords:** Mechanical valve, pannus, prosthetic valve obstruction, thrombus

## Abstract

**Background:**

Rheumatic heart disease is the most common cardiac disease in Yemen. It is associated with high morbidity and mortality. Valve replacement is the most common open heart surgery procedure in our cardiac center. The use of mechanical valves remains burdened with serious complications such as thrombosis. Valve thrombosis is still associated with high mortality^]^ The reported mortality rate of the redo operation ranges from 8 to 20% and up to 37-54% in critically ill patients.

**Objective:**

The aim of the present study was to investigate mechanical valve obstruction among Yemeni patients.

**Patients and Methods:**

Between January 2003 and April 2007, 2794 patients underwent prosthetic valve replacement in our center, Al-Thawra Hospital. Of those patients, 129 (4.6%) underwent reoperation for te obstructive mechanical valve. Patients with clinical suspicion of prosthetic valve obstruction (PVO) were admitted emergently to the CCU and the diagnosis was confirmed with echocardiography. All patients had heart failure; 95% of them were in NYHA class IV. All were transferred directly from the CCU to the operating room. The mean age was 34.8 ± 13.4 years. Two patients received preoperative thrombolytic therapy that was not successful. Obstruction involved the mitral valve prosthesis in 47 (36.4%); the aortic prosthesis in 16 (12.4%) patients; both valves in 21 (16.3%) patients; mitral valve replacement with tricuspid valve repair in 22(17%); double valve replacement with tricuspid valve repair in 1 (0.8%); redo mitral valve replacement with aortic valve cleaning in 7 (5.4%) cases; aortic valve cleaning in 5 (3.9%) patients; mitral valve cleaning in 5 (3.9%); and 5 (3.9%) patients had redo mitral with aortic replacement.

**Results:**

The operations were performed urgently. The etiology of the obstruction was thrombus in 111 (86%), pannus formation in 4 (3%), pannus and thrombus in 6 (4.8%), vegetation in 7 (5.4%) patients, and interposition of suturing materials in 1 (0.8%) patient. The in- hospital mortality was 23/129 (17.8%).

**Conclusion:**

The incidence of prosthetic valve obstruction remains high in Yemen. The vast majority of the patients who are referred to our hospital come from remote provinces in the country where regular INR measurement is not readily available. For those who are living in areas without good medical care, certain measures are necessary to avoid this disastrous complication: (1) good patient education, (2) free INR testing and free anticoagulant drugs such as warfarin; and (3) use of tissue rather than mechanical valves.

## INTRODUCTION

Rheumatic heart disease is the main cardiovascular disease in Yemen affecting children and young adults. The incidence of rheumatic fever and rheumatic heart disease is 1/1000 in the developing countries with high morbidity and mortality.

Intracardiac mechanical prosthetic valve replacement was first introduced in the 1960s, and many modifications in design and structural properties have improved the hemodynamic performance and durability of mechanical valves since then. However, prosthetic valve obstruction (PVO) continues to be a potential serious complication with a high risk of mortality. PVO is frequently related to thrombus formation due to inadequate anticoagulation; however, it can also be related to pannus formation due to overgrowth of fibrous tissue.[1] Prosthetic valve obstruction due to pannus or thrombus is related mainly to the surface character of the prosthetic valve and to the design and the material used in these valves as well as blood flow which depends on cardiac output, turbulence, stagnation, and the properties of the patient’s blood such as hypercoagulability.[2]

## METHODS

Between January 2003 and December 2007, 4783 operations were performed at the Cardiac Centre of Al-Thawra Hospital. A total of 2794 (58.4%) of these patients were operated upon for prosthetic valve replacement. This number includes valve replacements with or without coronary artery bypass or other cardiac procedures. One hundred twenty-nine of those patients presented with PVO. All patients were admitted as an emergency to the CCU with clinical suspicion of PVO. The diagnosis was confirmed by transthoracic echocardiography in all patients. Transesophageal echocardiography was performed in 24 patients (31%), and cineangiography in 10 patients (7.8%).

The obstruction of the prosthetic valve with pulmonary edema and NYHA class IV was present in 123 cases (95.3%), and 7 patients (4.7%) were in NYHA class III. Thrombolytic therapy was tried in two patients, which was not successful. Inadequate anticoagulation level as measured with International Normalized Ratio (INR) was found in most patients. An INR less than 2 was detected in 87 (67.4%) of the patients. The mean INR level in these patients was 1.43 ± 0.24.

All patients were transferred to the operating room and urgent redo operations were performed. The right femoral artery was cannulated in 12 (9.3%) patients. The right femoral artery and vein were cannulated in 7 patients (5.4%), and the ascending aorta was cannulated in 122 (94.6%) patients. Venous cannulation was performed bicavally, or by cannulating the superior vena cava together with the right femoral vein. A vent cannula was inserted into the right upper pulmonary vein for left ventricular venting in all patients. Antegrade hypothermic, blood, or crystalloid cardioplegia was administered for myocardial protection; 28-30°C systemic hypothermia was maintained. In some cases, where the mitral valve was exposed following the right atriotomy, the transseptal approach was preferred due to severe adhesions. The aortic cross-clamping time ranged from 29 to 190 min and the mean time was 85.5 ± 36.4 min, and total perfusion time (TPT) was 135.3 ± 68.73 min.

## RESULTS

**Figure 1:**
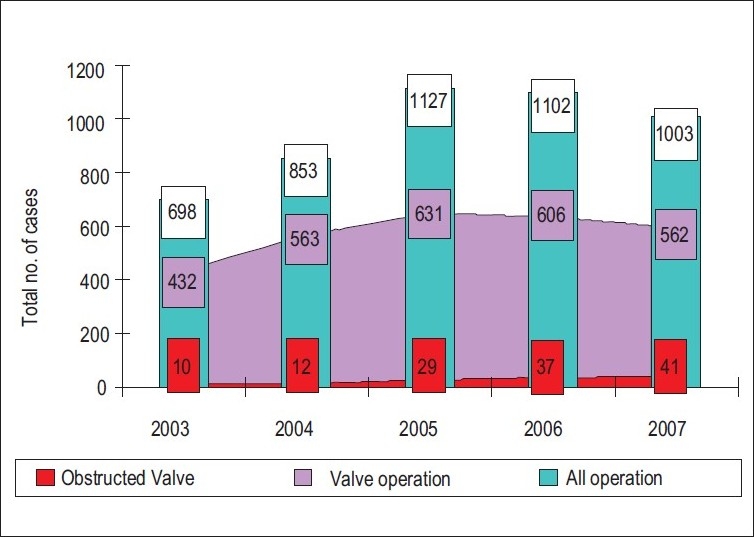
Incidence of obstructed valves are 4.9% from valves operations and 2.7% from total operations

**Figure 2:**
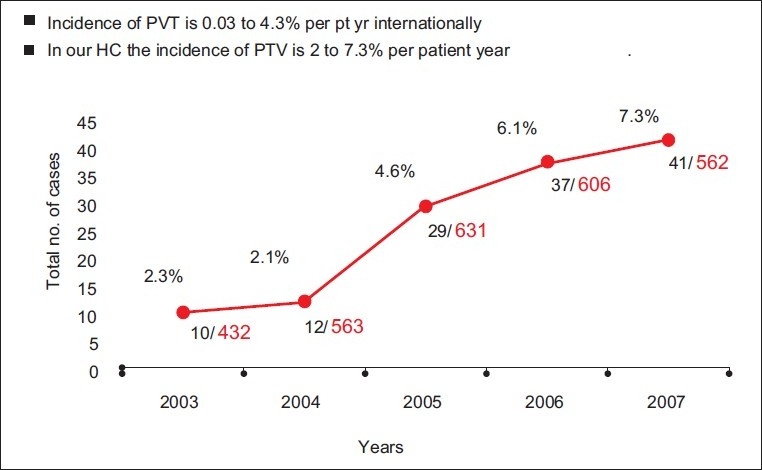
Obstructed valves per year

129 patients with PVO were studied retrospectively over 5 years (2003–2007). The incidence of PVO during this time is shown in Figures 1 and 2. The mean age of patients was 34.8 ± 13.4 (range, 10–70) years. Seventy-five patients (58%) were females and 54 patients (42%) were males. The mean time interval between first prosthetic valve implantation and valve dysfunction presentation was 2.2 ± 1.6 years (range, 4 days to 20 years).

**Figure 3:**
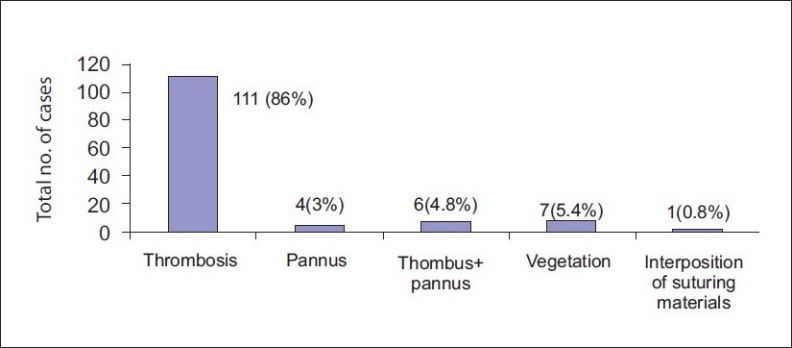
Causes of valve obstruction

The causes and incidence of prosthetic valve obstruction are shown in Figure 3. Thrombus was found in 111 (86%) patients, pannus formation in 4 (3%), pannus and thrombus in 6 (4.8%), vegetation in 7 (5.4%), and interposition of suturing materials in 1 (0.8%).

Prosthetic aortic valve obstruction was documented in 50 patients; 44 (88%) of them were bileaflet prostheses, 30 of which were St. Jude Medical, 3 CarboMedics, 10 Mira-Edwards, and 1 Sorin-Bicarbon; 4 (8%) were tilting-disc valves, and 2 (4%) were caged-ball valves.

There were 108 mitral prosthesis obstructions involving bileaflet valves in 102 (94.4%) of 108 patients (79 St. Jude Medical, 2 CarboMedics, 1 Sorin-Bicarbon, 20 Mira-Edwards), and tilting-disc valves in 6 (5.6%) patients.

Of the 129 patients with proven prosthetic valve obstruction, 47 (36.4%) patients underwent mitral valve surgery, 16 patients (12.4%) had aortic valve surgery, and 21 (16.3%) had both aortic and mitral valve operations. Twenty-two (17%) patients had mitral valve replacement with tricuspid valve repair, 1 (0.8%) had double valve replacement (DVR) with tricuspid valve repair, 7 (5.4%) had redo mitral valve replacement with aortic valve cleaning, 5 (3.9%) had aortic valve cleaning, 5 (3.9%) had mitral valve cleaning, and 5 (3.9%) had redo double valve replacement [Table 1].

**Table 1:** Procedures and implanted substitute prosthetic valves

Procedure	Mitral	Aortic	TV
Debridement	5	12	–
Repair	–	–	21
Replacement	103	38 + 5 new implantations	2
Bioprosthesis	6	2	2
Bileaflet	97	41	–
CarboMedics	3	2	–
Mira-Edwards	10	3	–
St. Jude	84	35	–
Sorin-Bicarbon	–	1	–

Intensive Care Unit (ICU) stay was between 4 and 22 days. Eighty-two patients were transferred from ICU to the ward without complications. Forty-seven patients had complications during hospitalization; two patients had bleeding; CVA developed in four patients, and two of them expired. Five patients developed renal failure which required dialysis, three improved, and two expired with multiorgan failure. Nine patients had prolonged intubations due to severe chronic obstructive pulmonary disease (COPD), three of them required tracheostomy; five patients expired due to multiorgan failure and sepsis. Two patients developed hepatic impairment and were managed successfully. 18 patients developed cardiogenic shock, 15 of them expired. Two cases were complicated and one case expired due to other causes. The total in-hospital mortality rate was 17.2% (23 patients).

## DISCUSSION

Rheumatic heart disease is the main cardiovascular disease in Yemen and it is associated with a high morbidity and mortality. Valvular replacement with a mechanical valve is the main therapy for advanced valvular heart disease. Mechanical valves need lifelong anticoagulation therapy. The increased incidence of valve dysfunction observed in this series can be explained by the increased number of valve replacement operations performed at our cardiac center year by year as shown in [Figure 2]. The main causes of the observed high incidence of prosthetic valve obstruction are poverty and lack of adherence of patients to medical instructions especially anticoagulant therapy.

Thrombus formation is due to the interaction of cellular and plasmic components of blood with injured endocardium, structural and metabolic changes due to variable blood flow, and the surface of the mechanical valve that has thrombogenic properties.[3,4] Thrombotic and thromboembolic events seen in the first months following surgery can be explained by an increased activity of the intrinsic coagulation system due to contact with the nonendothelialized surface of the cuff of the mechanical valve and the presence of injured tissues.[5]

Our protocol for anticoagulation therapy is to keep the therapeutic level of INR between 2 and 3.5 in patients undergoing mitral or aortic mechanical valve replacement, and between 2.5 and 4 in those undergoing double mechanical valve replacements. Patients receive information regarding the importance of anticoagulation for their prosthetic valves and for their lives. Warfarin dose is adjusted before discharge to reach the desired INR level. Patients are asked to come back to the cardiac center 2 weeks after discharge. However, the vast majority of the patients are referred to our hospital from other provinces of the country, where a regular INR measurement is usually not available. Therefore, it is impossible to say exactly whether anticoagulation is kept within therapeutic range in all patients in the follow-up period after 2 months. Most of the patients who live in towns and villages where there are no facilities to check the INR are probably not following the required level. Others may be not taking anticoagulation either because of poverty, or because they do not follow the medical instructions; still, others may not be monitoring their INR and consequently, inadequate anticoagulation levels were detected in 67.4% (87 of 129) of patients in this study.

Thrombus formation on the valves is a condition stimulated by thrombocyte activation and factors such as the adequacy of anticoagulation, cardiac rythym, and blood flow characteristics[1,6]

Pannus formation, which is an overgrowth of the fibrous tissue, is an inflammatory foreign body reaction that may invade the valve orifice Histological investigations showed that collagen and elastic tissues contain endothelial cells, chronic inflammatory cells, and myofibroblasts in pannus tissue.[7] Pannus tissue can grow very slowly and may cause obstruction within 10–15 years after mechanical valve replacement.[5] A thrombus layer can be secondarily formed on pannus. In this study, we detected thrombus in 111 of 129 (86%) patients and pannus in 4 of 129 (3%), pannus and thrombus in 6 of 129 (4.8%), and vegetations in 7 of 129 (5.4%) patients. Compared with published studies, thrombus formation was quite high among our patients (86%). Deviri and colleagues reported 77.7% thrombus formation, pannus in 10.7%, and pannus and thrombus in 11.6% among 112 patients who they studied.[1]

In a study by Devies *et al*.,[8] the most common site of thrombus formation was the atrial side of the mitral valve. Turbulent flow, recirculation areas, and transprosthetic flow conditions result in high risk of thrombus formation in the atrioventricular position as compared to aortic.[8] Lower pressure areas cause the formation of the more fibrous tissue. Abnormal formation of the fibrous tissue, which plays a role in surrounding cuff epithelialization according to the location of the prosthetic valve, is most commonly seen on the atrial side of the tricuspid valve followed by the atrial side of the mitral valve and ventricular side of the tricuspid valve. Because of the high pressure, a thin layer of endothelial tissue formation occurs on the ventricular side of the mitral valve and on both sides of the aortic valve. These findings can explain why thrombotic events are more common in the tricuspid and mitral as compared to the aortic position.[2,9,10]

All patients underwent emergency surgery. All factors transiently or permanently stimulating the coagulation factors and thrombocyte aggregation increase the risk of local thrombus formation. The level of coagulability is determined by the instant effects of procoagulant and anticoagulant factors. Inadequate anticoagulation, loss of atrial contraction, oral contraceptives, systemic drugs, such as estrogen, systemic diseases, such as malignant tumors, a defect on the surface of a mechanical prosthetic valve, and chronic endothelial injury due to variable blood flow can all cause local hypercoagulability.[11,12] Thrombus on the mechanical valve may lead to congestive heart failure and CVA. Rarely, it may cause vasospastic angina due to coronary thromboembolism.[13]

The mortality rate in PVO is greater in cases with thrombus than with pannus. This is related to rapid hemodynamic deterioration associated with valve thrombosis.[14] Acute thrombus is less common in bileaflet than in tilting disk mechanical prosthetic valves.[15] However, in our study, PVO was observed in 117 of 129 (90.7%) patients with bileaflet mechanical valves.

Nonsurgical management such as thrombolytic therapy can also be administered to patients with prosthetic valve obstruction due to thrombus formation.[16] In this study, two patients received thrombolytic treatment preoperatively but thrombolysis failed in both patients. The transvalvular gradient remained high and the patients had to undergo cardiac surgery.

## CONCLUSION

Prosthetic valve dysfunction is an emergency condition with hemodynamic deterioration and high mortality in valvular reoperations. The high incidence of valve thrombosis among Yemeni patients necessitates urgent steps to find solutions to avoid this disastrous complication. For those who are living in areas without good medical care, there are a number of measures that could be taken: (1) good patient education pre- and postoperation; (2) provision of free anticoagulation drugs such as warfarin; (3) closer attention to INR level monitoring could be achieved with the distribution of free INR kits; and (4) use of tissue valves rather than mechanical valves. All these may be good solutions to address this problem.
